# Feeding Difficulty Among Chinese Toddlers Aged 1–3 Years and Its Association With Health and Development

**DOI:** 10.3389/fped.2021.758176

**Published:** 2021-11-23

**Authors:** Zhongxia Ren, Hanglian Lan, Ignatius Man-Yau Szeto, Chenlu Yang, Jian Zhang, Pin Li, Jingwen Li, Peiyu Wang, Yumei Zhang, Ai Zhao

**Affiliations:** ^1^Department of Nutrition and Food Hygiene, School of Public Health, Peking University Health Science Center, Beijing, China; ^2^Yili Maternal and Infant Nutrition Institute, Inner Mongolia Yili Industrial Group Co., Ltd., Hohhot, China; ^3^Department of Social Science and Health Education, School of Public Health, Peking University Health Science Center, Beijing, China; ^4^Vanke School of Public Health, Tsinghua University, Beijing, China

**Keywords:** feeding difficulty, the Montreal Children's Hospital Feeding Scale, toddlers, health and development, the Ages and Stages Questionnaire

## Abstract

Feeding problems are biopsychosocial in nature and have a great influence on children's growth. The aim of this study was to profile the status and possible influencing factors of feeding difficulty among normal Chinese toddlers, and to investigate its association with health and development. This study is a part of the Young Investigation (YI study) conducted in 10 cities in China. Data from 924 children aged 1–3 years were analyzed. Data on socio-demographic factors, feeding behaviors, self-reported diseases, and anthropometry parameters were collected. Blood samples were drawn to determine hemoglobin levels. Feeding difficulty was evaluated by the Montreal Children's Hospital Feeding Scale (MCH-FS). Ages and Stages Questionnaires, Third Edition (ASQ-3) were used to assess developmental progress. Multivariable analyses were performed to explore the potential associations. The mean total score of the MCH-FS was 35.21 ± 12.90 and the highest scored item was “acting up/making a big fuss during mealtimes.” Feeding difficulty occurred more often among children with picky eating behavior or whose caregivers once used the strategy of pre-mastication. Children with feeding difficulty had lower intakes of cereals, vegetables, and fruits, and were more likely to suffer from diarrhea (*OR*, 2.04; *95%CI*: 1.32, 3.11) or constipation (*OR*, 2.04; *95%CI*: 1.27, 3.24), but not anemia. Feeding difficulty was also negatively associated with weight, height, head circumference and mid-upper-arm circumference-related Z-scores (*P* all < 0.05). In addition, it was related to poorer fine motor skills, personal and social skills, and total scores of ASQ-3 (*β*, −9.00; *95%CI*: −15.11, −2.89). Feeding difficulty assessed by MCH-FS showed a negative association with children's health and development, supporting the need for early identification.

## Introduction

It is well-established that toddlerhood is a crucial period for the establishment and consolidation of eating behaviors. During this transitional period, feeding problems are common and merit particular attention. According to the framework of the World Health Organization (WHO) International Classification of Functioning, Disability, and Health (ICF), a unifying diagnostic term “pediatric feeding disorder (PFD)” was proposed and defined as “impaired oral intake that is not age-appropriate, and is associated with medical, nutritional, feeding skill, and/or psychosocial dysfunction” (1). However, the symptoms of feeding-related problems are heterogeneous, including oral motor delays, dysphagia, poor appetite, food refusal, or selectivity, self-feeding inadequacy, excessive mealtime duration, acting up during mealtime, or other inappropriate behaviors (1–3). In addition, it is generally accepted that feeding problems are biopsychosocial in nature (4), and their possible explanation include medical factors (e.g., developmental and neurological disabilities), infant temperamental and psychological factors, parental feeding styles, and interactions between parent and child, as well as environmental factors (1, 3, 5). Thus, a thorough assessment and identification is no simple task.

Notably, compared with the serious feeding problems that occur in children with a known medical etiology, feeding problems among normal children are softer but more difficult to assess (2, 6), with a prevalence of around 7–45% worldwide (3, 7, 8). On the one hand, caregivers may not be aware of this kind of feeding problem as it is sometimes indistinguishable from the temporary imperfect behaviors during the development stage (9). On the other hand, the impact may be too small to be noticed in a short time. However, feeding problems in early childhood, if unaddressed, might track into later adolescence and even adulthood (7, 10, 11). A small problem persisting for a prolonged time may also significantly impact a child's nutritional status, physical growth and cognitive development, and cause stress to caregivers (2, 3). For example, Pan et al. found that consumption of sugar-sweetened beverages by infants increased the risk of obesity at 6 years of age (12). Another longitudinal study conducted in 181 non-Hispanic white girls indicated that picky behavior showed a certain degree of continuity from ages 5 to 15 years, and persistent picky eaters had significant lower BMI than did non-picky eaters (13). However, feeding practices have been identified as one modifiable determinant and several systematic reviews have showed that behavioral interventions would significantly improve feeding behavior (14, 15). As a result, an easy but valid tool is needed for cares and pediatricians to enable early identification and rapid intervention, which will help to optimize children's eating skills and avoid negative consequences.

Several standardized questionnaires have been developed as screening instruments to assess feeding problems, which are often long or require administration by a clinician (2, 10). Instead, a quick but reliable screening tool, the Montreal Children's Hospital Feeding Scale (MCH-FS), was developed by Ramsay et al. (4). It contains only 14 items and enables the rapid identification of feeding difficulty (approximately 5 min). This stool is suitable for children of a wide range of ages, from 6 months to 6 years old and is primarily based on parent report, which can offer a “holistic” perspective efficiently (3). Further, it has been confirmed to have excellent construct validity and reliability (4, 16–21).

Currently, the MCH-FS was primarily used to assess feeding difficulty in children with risk factors, such as premature (3), esophageal atresia (EA) (6, 22, 23), agenesis of the corpus callosum (ACC) (24), congenital heart disease (25), asthma (26), Down's syndrome (27), and autism spectrum disorder (ASD) (28), but less is known about the association between MCH-FS and health and development in the normal population. However, the discrimination score for identifying feeding problems set by Ramsay et al. was obtained by the normative sample recruited from the community pediatricians' offices (4). In a subsequent study, Rogers et al. applied the MCH-FS among 69 mother–infant pairs recruited on postnatal low-risk wards in Birmingham, United Kingdom at 1 year after birth, and they demonstrated that the MCH-FS showed significant overlap with other measures of feeding and was associated with the baby weight across the first year (10). Thus, the MCH-FS might be a potentially useful tool for researching feeding problems in community samples without other risk factors.

Given the burden on primary care services, this study's aims were to profile the status and possible influencing factors of feeding difficulty among Chinese toddlers without a basic medical etiology, and to investigate its association with health and development.

## Materials and Methods

### Subjects

This study was based on a cross-sectional survey focused on the nutrition and health of pregnant women, mother–infant pairs, and toddlers (Young Investigation, also named YI study), which was conducted in 10 cities of China from 2019 to 2020. In the section of toddlers, a multistage sampling strategy was adopted to draw the sample. First, two first-tier cities (Beijing and Guangzhou) and eight non-first-tier cities (Chengdu, Hohhot, Lanzhou, Nanchang, Ningbo, Shenyang, Suzhou, and Xuchang) were purposely selected based on diverse characteristics of geographical location and socio-economic status, of which five (Chengdu, Guangzhou, Nanchang, Ningbo, and Suzhou) are located in southern and the others in northern China (Beijing, Hohhot, Lanzhou, Shenyang, and Xuchang). In the second step, one hospital or maternal and child healthcare facilities was selected in each city using the convenience sampling method. Children were conveniently recruited according to their visiting time until the number of completed participants reached the target of 90 per city. The inclusion criteria comprised singleton, full-term (gestational age ≥37 weeks), children aged 1–3 years. Exclusion criteria comprised children with physical disabilities, infectious diseases, mental diseases, and other major diseases (metabolic-related diseases, etc.) or with guardians who could not answer the questions. Based on the calculation formula of sample size for cross-sectional study:


$$\begin{array}{l} n=\frac{Z_{1-\alpha /2}^{2}*p\left(1-p\right)}{d^{2}} \end{array}$$

Where *n* was sample size required, α was significant level set at 0.05, *p* was estimated prevalence of feeding difficulty set as 0.2 according to a previous study conducted in China (29), *d* (admissible error) was 0.15*p* here, the minimum theoretical sample size was calculated as around 700. Finally, a total of 924 individuals were enrolled and all of them were included, satisfying the calculated sample size requirements.

This study was conducted according to the guidelines of the Declaration of Helsinki, and approved by the Ethics Committee of Peking University (NO. IRB00001052-19045). All parents/guardians were informed and signed the informed consent.

### Data Collection

#### Sociodemographic and Lifestyle Factors

A uniform paper questionnaire was administered to parents/guardians by trained investigators to capture children's socio-demographic information (age, sex, per capita monthly income, mother's education level, mother's occupation, place of residence, and birth weight), outdoor activity, feeding-related characteristics (primary caregivers, timing of introduction of first supplementary food, duration of exclusive breastfeeding, picky eating behaviors, pre-mastication, and food allergy), and self-reported diseases (respiratory diseases, allergic diseases, vomiting, diarrhea, and constipation within the previous 3 months).

Children's dietary intakes over the past month were assessed by parents/guardians using a 53-item semi-quantitative food frequency questionnaire (FFQ) with the help of standard measuring bowls, cups, spoons, and pictorial food atlas. According to the Chinese Food Composition Table (30), all food items were categorized into 11 food groups including dairy products (ml), cereals (g), potatoes (g), soybean and nuts (g), vegetables (g), fruits (g), meat and poultry (g), aquatic products (g), eggs (g), beverages (ml), and snacks (g), and daily intakes for each group were calculated. In addition, the daily dietary intakes of energy, protein and the dietary diversity score (DDS) was assessed using a 24-h dietary recall. Based on the Chinese dietary guidelines (CDG) for children (31), the following nine food groups were included to calculate the DDS: dietary products, cereals and potatoes, soybean and nuts, vegetables and pure vegetable juice, fruits and pure fruit juice, meat and poultry, aquatic products, eggs, and moderation foods (including snacks, cakes, beverages, etc.) (32). If a participant consumed any food within the above nine groups in the past 24 h, then he/she would get one point for a certain food group. The food groups were not counted repeatedly, thus the DDS ranged from one to nine.

#### The Montreal Children's Hospital Feeding Scale

The MCH-FS contains 14 items based on the biopsychosocial model, covering the following domains: parental concerns about feeding and children's growth; strategies (use of distractions or force); children's oral motor and sensory (gagging/spitting or vomiting, holding of food in the mouth, chewing/sucking); appetite, mealtime duration, and behaviors (acting up/making a big fuss); and family relationships influenced by feeding (4, 16). The Chinese version of the MCH-FS was used in this study to identify children's feeding problems. All 14 items were retained within the Chinese version, and it has good validity and reliability (21). Parents/caregivers were asked to respond to each item based on a seven-point Likert scale (range, 1–7), of which seven items were scored from the negative to positive direction and the others were with reverse direction. The total score (range, 14–98) was calculated by summing up the scores for all the 14 items after reversing the scores of the seven items whose scales were from negative to positive (seven minus the raw score). The higher the total score indicated the more severe the symptoms of feeding difficulties. The discrimination score for identifying feeding difficulty was set as one standard deviation above the mean total score (4).

#### The Ages and Stages Questionnaires, Third Edition

Children's developmental progress was measured by the Ages and Stages Questionnaire, third edition (ASQ-3) (33). The ASQ-3 is a well-recognized developmental screening tool and has been translated and adapted into a Simplified Chinese version (34). The questionnaires were designed to assess five developmental domains in children aged 1 month to 5.5 years: communication (CM), gross motor skills (GM), fine motor skills (FM), problem-solving ability (cognition, CG), and personal and social skills (PS). Each domain includes six questions based on relevant skills, each of which is answered on a three-point scale as yes (scored 10), sometimes (scored 5), or not yet (scored 0). Thus, the total score range of every domain is 0–60 and higher scores indicated better development. The questionnaire was completed by children's parents/guardians with the aid of medical staff and scored automatically using an online ASQ calculator.

#### Anthropometric Measurements and Growth Assessment

Anthropometric measurements consisting of height (cm), head circumference (cm), mid-upper arm circumference (cm), and weight (kg) were taken by uniformly trained investigators using standard protocols and tools, with precision to one decimal place. For all the measurements, the children were standing and wore light clothing without shoes. The WHO Anthro software (version 3.2.2) was used to calculate the anthropometric Z-scores, including: weight-for-height Z-score (WHZ); weight-for-age Z-score (WAZ); height-for-age Z-score (HAZ); body mass index-for-age Z-score (BAZ); head circumference-for-age Z-score (HCZ); and mid-upper arm circumference-for-age Z-score (MUACZ).

#### Detection of Peripheral Blood Hemoglobin

Children's fingertip blood samples were collected through pricking the left-hand middle fingertips by nurses, and the blood spots were instantly put onto CompoLab TS portable hemoglobin analyzer (CompoLab TS, DiaSpect Medical GmnH, Germany) to detect the hemoglobin (Hb) concentration. According to the recommendations of WHO (35), the Hb concentrations were calibrated for altitude, and a cut-off of 110 g/L was used to define anemia.

### Statistical Analysis

R software (version 4.1.0) was used for statistical analysis. Categorical variables were presented as numbers (percentage) and continuous variables were described as mean (standard deviation) or median (25th percentile, 75th percentile) according to the normality. Participants were divided into two groups as with or without feeding difficulty, and independent samples *t*-test or chi-squared tests were used to evaluate the differences in MCH-FS scores and socio-demographic characteristics between the two groups. To explore possible influence factors on feeding difficulty, binary logistic regressions analyses were performed. In addition, using children without feeding difficulty as the reference group, multivariate linear or logistic regressions were carried out to analyze the association between feeding difficulty and children's dietary intakes, anthropometric Z-scores, illness occurrences in recent 3 months and ASQ-3 scores. Mediation effects of feeding difficulty through daily energy or protein intakes on health and development measurements were conducted using the R package “mediation” (36). A series of prior confounding factors were adjusted in the multivariate analysis, including age, sex, mother's education level, monthly household economic level, outdoor activity level, place of residence, and birth weight. The significance level for all the statistical tests was set at 0.05.

## Results

### Individual Item and Total Scores of the MCH-FS

**Figure 1:**
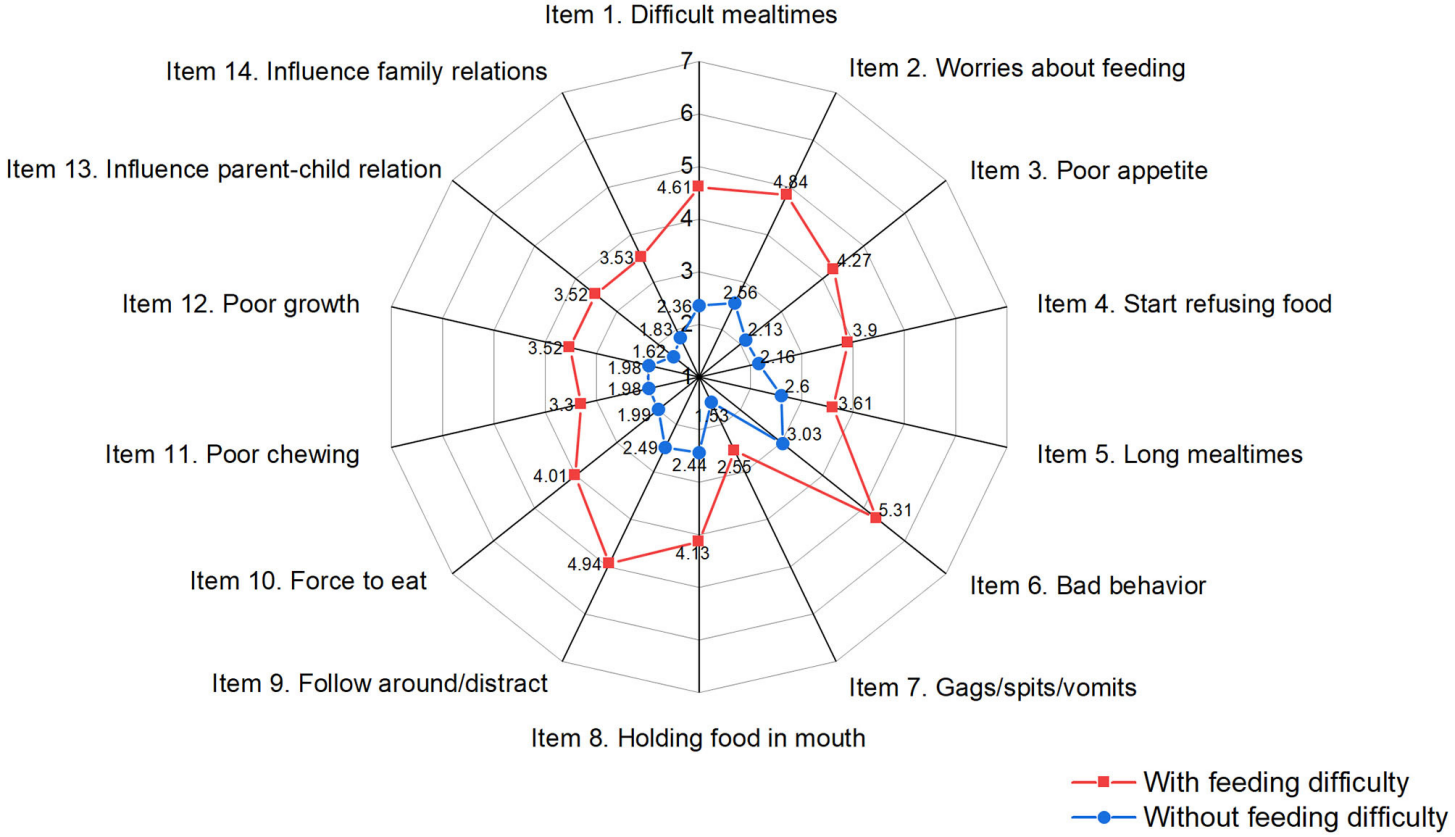
Mean score of the 14 items of MCH-FS based on classification of feeding difficulty.

The mean and standard deviations of individual items and total scores of the MCH-FS are shown in Figure 1 and Supplementary Table 1. The mean total score of MCH-FS was 35.21 ± 12.90. Of all the 14 items, the highest scored item was “bad behavior during mealtimes (such as acting up/making a big fuss)” and the lowest was “Gagging/spitting/vomiting with certain types of food.” A total score of 48.11, which is 1 SD above the mean score was used as the discrimination score, and 18% (165/924) of all the children were identified with feeding difficulty. The children with feeding difficulty obtained significantly higher scores for all individual items as well as the total scores in comparison to those without feeding difficulty.

### Socio-Demographic Characteristics of Children With or Without Feeding Difficulty

Table 1 provides an overview of the socio-demographic characteristics of children with or without feeding difficulty. Children with feeding difficulty were older, had a higher proportion of girls and spent less time in outdoor activities than children without feeding difficulty. The distribution of the place of residence was also significantly different between the two groups. However, the differences in a series of natal factors (delivery mode, parity, birth weight) or maternal factors (per capita monthly income level, mother's education level, mother's occupation) between the two groups were not significant.

**Table 1:** Socio-demographic characteristics of children with or without feeding difficulty.

**Variable**	**Feeding difficulty** ^a^		***P*** ^b^
	**With** **(***n*** = 165)**	**Without** **(***n*** = 759)**	
**Age (months)**			**<0.001**
~18	34 (20.6)	225 (29.7)	
~24	41 (24.8)	212 (28.0)	
~30	50 (30.3)	130 (17.2)	
~36	40 (24.2)	190 (25.1)	
**Sex**			**0.032**
Boy	71 (43.3)	400 (52.9)	
Girl	93 (56.7)	356 (47.1)	
**Delivery mode**			0.408
Cesarean delivery	67 (40.6)	336 (44.5)	
Vaginal delivery	98 (59.4)	419 (55.5)	
**Parity**			0.131
1	111 (67.3)	460 (60.6)	
>1	54 (34.6)	299 (39.4)	
**Birth weight (kg)**	3.41 ± 0.70	3.41 ± 0.52	0.959
**Per capita monthly income (RMB: yuan)**			0.935
~3,000	28 (17.5)	132 (17.7)	
~5,000	42 (26.2)	213 (28.6)	
~10,000	59 (36.9)	264 (35.4)	
>10,000	31 (19.4)	136 (18.3)	
**Mother's education**			0.424
Middle school or below	93 (12.3)	26 (15.8)	
High school or equal	341 (45.0)	68 (41.2)	
College or above	323 (42.7)	71 (43.0)	
**Mother's occupation**			**0.339**
Unemployed	10 (6.1)	27 (3.6)	
Office work	78 (47.6)	402 (53.3)	
Manual work	39 (23.8)	172 (22.8)	
Other work	37 (22.6)	153 (20.3)	
**Place of residence**			**0.024**
Beijing	11 (6.7)	80 (10.5)	
Chengdu	15 (9.1)	77 (10.1)	
Guangzhou	27 (16.4)	63 (8.3)	
Hohhot	10 (6.1)	82 (10.8)	
Lanzhou	16 (9.7)	74 (9.7)	
Ningbo	18 (10.9)	78 (10.3)	
Nanchang	23 (13.9)	71 (9.4)	
Shenyang	17 (10.3)	75 (9.9)	
Suzhou	16 (9.7)	74 (9.7)	
Xuchang	12 (7.3)	85 (11.2)	
**Outdoor activity (h/day)**			**0.018**
≤1	36 (22.1)	107 (14.3)	
>1	127 (77.9)	643 (85.7)	

*Bold text represents a statistically significant difference (p < 0.05)*.

a *Values are presented as frequency (percentage) or mean ± standard deviation*.

b *P-values were obtained from chi-squared tests or independent samples t-test*.

### Factors Associated With Feeding Difficulty

A series of factors potentially related to feeding difficulty were analyzed (Table 2). The results from logistic regression show that feeding difficulty occurred more often among children with picky eating behavior or whose caregivers once used the strategy of pre-mastication. No statistically significant association was observed for primary caregivers, timing of introduction of first supplementary food, duration of exclusive breastfeeding, or presence of food allergy with feeding difficulty.

**Table 2:** Association between feeding-related characteristics and feeding difficulty.

**Variable**	**Feeding difficulty** ^a^		**Unadjusted**	**Adjusted**
	**With**	**Without**	***OR (95% CI)***	***OR (95% CI)*** ^b^
**The primary caregivers**				
Parents	95 (57.6)	422 (55.6)	1.00 (Ref.)	1.00 (Ref.)
Grandparents or others	70 (42.4)	337 (44.4)	0.846 (0.601, 1.188)	0.875 (0.588, 1.297)
**Timing of introduction of first supplementary food (months)**			
<6	40 (25.2)	150 (20.2)	1.00 (Ref.)	1.00 (Ref.)
≥6	119 (74.8)	591 (79.8)	0.755 (0.509, 1.137)	0.881 (0.569, 1.387)
**Duration of exclusive breastfeeding (months)**				
Never	38 (24.4)	136 (18.6)	1.00 (Ref.)	1.00 (Ref.)
<6	29 (18.6)	152 (20.8)	0.682 (0.397, 1.164)	0.695 (0.389, 1.234)
≥6	89 (57.1)	443 (60.6)	0.719 (0.473, 1.109)	0.729 (0.457, 1.180)
**Picky eater**				
No	66 (40.2)	525 (69.2)	1.00 (Ref.)	1.00 (Ref.)
Yes	98 (59.8)	234 (30.8)	**3.331 (2.357, 4.734)**	**3.351 (2.288, 4.942)**
**Pre-mastication**				
No	132 (80.0)	679 (89.7)	1.00 (Ref.)	1.00 (Ref.)
Yes	33 (20.0)	78 (10.3)	**2.176 (1.377, 3.380)**	**2.405 (1.445, 3.946)**
**Food allergy**				
No	134 (81.2)	648 (85.6)	1.00 (Ref.)	1.00 (Ref.)
Yes	31 (18.8)	109 (14.4)	1.375 (0.874, 2.114)	1.388 (0.847, 2.224)

*Bold text represents a statistically significant difference (p < 0.05)*.

a *Values are presented as frequency (percentage)*.

b *Adjusted for age, sex, mother's education level, household economic level, outdoor activity level, place of residence, and birth weight*.

### Difference of Dietary Intakes of Children With or Without Feeding Difficulty

In terms of the daily dietary intake over the past month, children with feeding difficulty had lower intake of cereals, vegetables and fruits, as exhibited in Table 3. Other than this, the daily intake of dairy products, potatoes, soybean and nuts, meat and poultry, aquatic products, eggs, beverages, and snacks were not significantly different between the two groups.

**Table 3:** Difference of dietary intakes of children with or without feeding difficulty.

**Daily dietary intake**	**Feeding difficulty** ^a^		**Unadjusted**	**Adjusted**
	**With**	**Without**	***β (95% CI)***	***β (95% CI)*** ^b^
Dairy products (ml)	403.0 (246.0, 600.0)	410.0 (203.5, 600.0)	19.230 (−37.070, 75.529)	16.814 (−43.628, 77.256)
Cereals (g)	58.0 (24.0, 114.0)	69.0 (30.0, 150.0)	**−22.411 (−40.143, −4.678)**	**−24.051 (−41.785, −6.316)**
Potatoes (g)	5.0 (1.0, 13.0)	9.0 (3.0, 20.0)	**−5.597 (−9.772, −1.421)**	−3.499 (−7.250, 0.253)
Soybean and nuts (g)	4.0 (1.0, 9.9)	4.3 (1.0, 13.0)	−2.059 (−5.392, 1.814)	−1.539 (−5.688, 2.610)
Vegetables (g)	32.0 (9.0, 73.0)	46.0 (18.0, 104.0)	**−18.303 (−34.438, −2.168)**	**−18.802 (−35.654, −1.950)**
Fruits (g)	100.0 (40.0, 160.0)	100.0 (50.0, 200.0)	**−30.673 (−53.368, −7.978)**	**−27.041 (−49.588, −4.493)**
Meat and poultry (g)	28.0 (11.0, 59.0)	31.0 (12.0, 66.0)	−5.625 (−13.918, 2.667)	−5.952 (−14.439, 2.534)
Aquatic products (g)	6.0 (2.0, 17.0)	8.0 (2.0, 22.0)	−4.164 (−9.890, 1.561)	−4.976 (−10.869, 0.916)
Eggs (g)	43.0 (17.0, 60.0)	50.0 (20.0, 60.0)	−1.796 (−7.162, 3.570)	−1.070 (−6.710, 4.570)
Beverages (ml)	401.0 (220.0, 600.0)	423.0 (224.5, 620.5)	−55.920 (−138.786, 26.940)	−20.525 (−98.154, 57.104)
Snacks (g)	10.0 (3.0, 22.0)	11.0 (3.5, 25.0)	−5.111 (−11.728, 1.506)	−6.444 (−13.627, 0.739)

*Bold text represents a statistically significant difference (p < 0.05)*.

a *Values are presented as median (25th percentile, 75th percentile)*.

b *Adjusted for age, sex, mother's education level, household economic level, outdoor activity level, place of residence, and birth weight*.

### Association Between Feeding Difficulty and Health and Development

**Figure 2:**
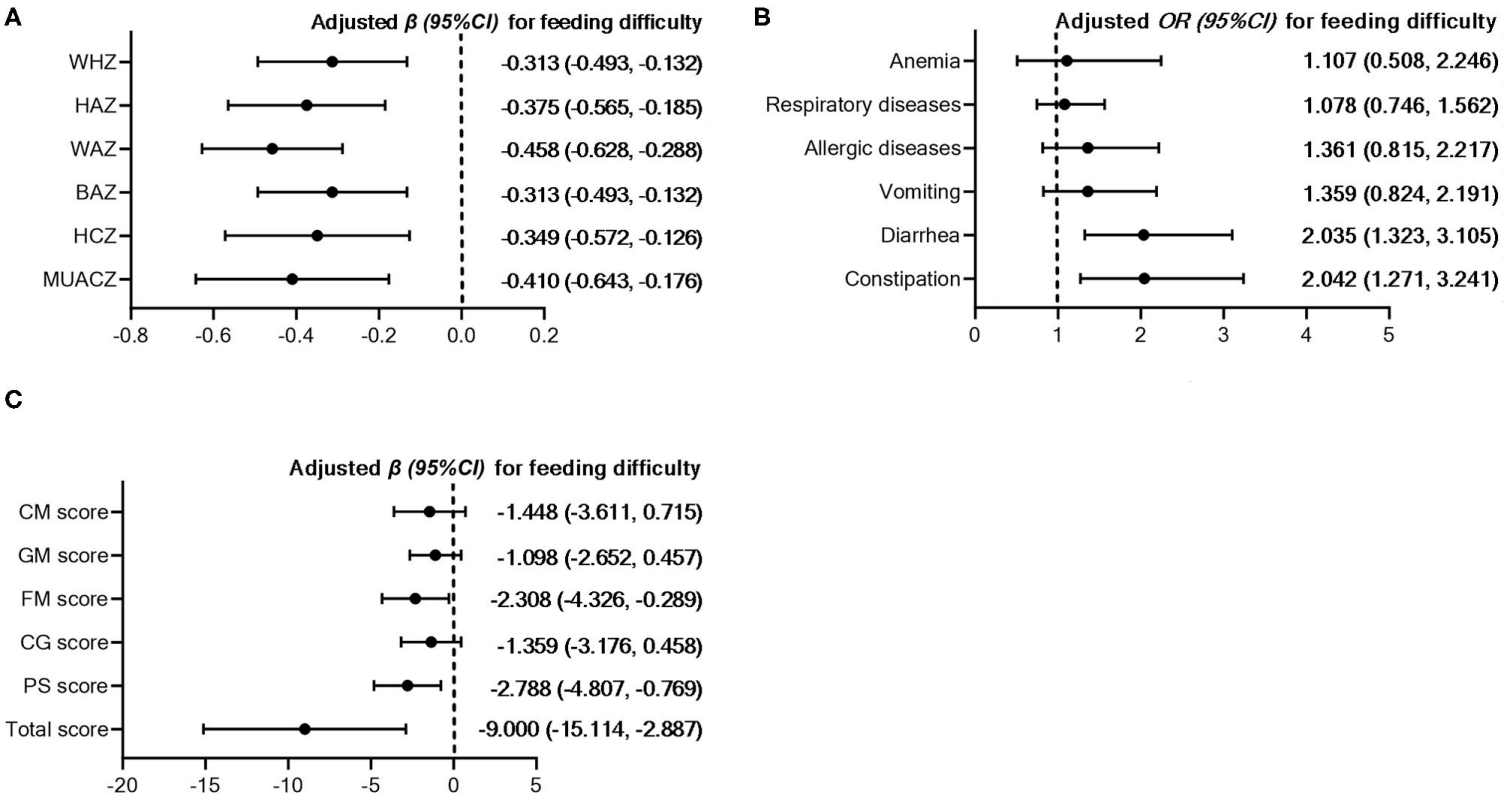
Association between feeding difficulty and anthropometric Z-scores **(A)**, illness occurrences in recent 3 months **(B)** and ASQ scores **(C)**. WHZ, weight-for-height Z-score; WAZ, weight-for-age Z-score; HAZ, height-for-age Z-score; BAZ, body mass index-for-age Z-score; HCZ, head circumference-for-age Z-score; MUACZ, mid-upper arm circumference-for-age Z-score; CM, communication; GM, gross motor skills; FM, fine motor skills; CG, cognition; PS, personal and social skills. Adjusted β (95%CI) or OR (95%CI) were obtained from multivariate linear or logistic regressions using children without feeding difficulty as the reference group. The regressions were adjusted for age, sex, mother's education level, household economic level, outdoor activity level, place of residence, and birth weight.

The associations between feeding difficulty and health and development are presented in Figure 2 and Supplementary Tables 2–4. Feeding difficulty was negatively associated with all the anthropometric Z-scores including WHZ, HAZ, WAZ, BAZ, HCZ, and MUACZ. When it comes to illness occurrences in recent 3 months, children with feeding difficulty were more likely to suffer from diarrhea or constipation, but not anemia, respiratory diseases, allergic diseases, or vomiting. In addition, feeding difficulty was also related to poorer fine motor, personal-social, and total scores of ASQ-3.

### Mediation Effects of Feeding Difficulty Through Daily Energy and Protein Intakes on Health and Development

**Figure 3:**
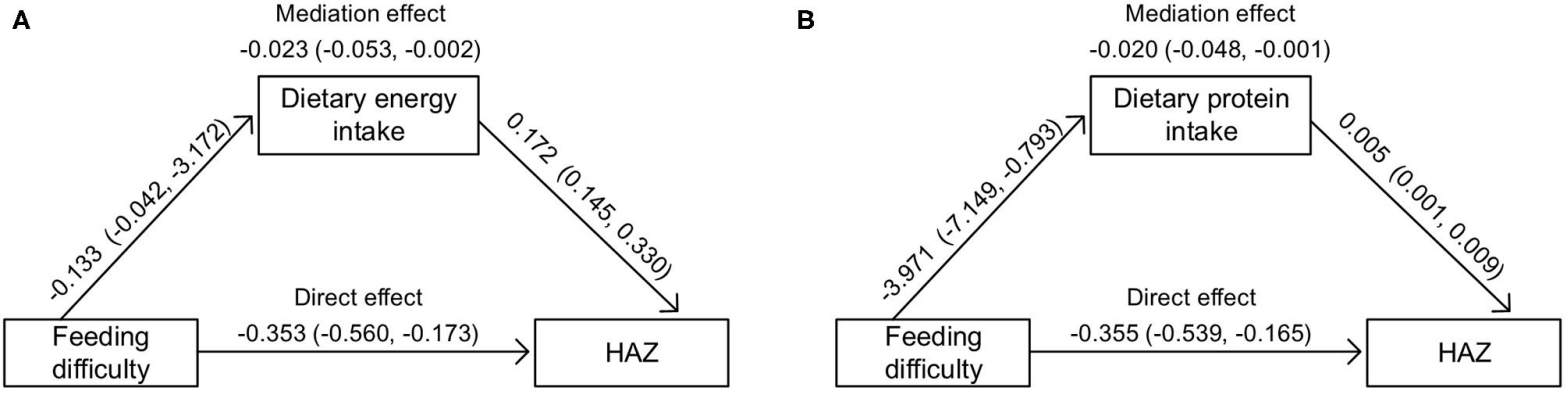
Mediation effects of feeding difficulty through daily intakes of energy [per 1,000 kcal, **(A)**] or protein [per g, **(B)**] on HAZ. HAZ, height-for-age Z-score. The regressions were adjusted for age, sex, mother's education level, household economic level, outdoor activity level, place of residence, and birth weight.

According to the dietary recall of the preceding day of the survey, children with feeding difficulty reported lower intakes of energy and protein, while the difference in DDS was not significant (Supplementary Table 5). In further mediation analysis, significant mediation effects of dietary intakes of energy or protein on the association between feeding difficulty and HAZ were observed (Figure 3). For the other anthropometric Z-scores or ASQ-3 scores, the mediation effects were not significant.

## Discussion

In this study, we assessed feeding difficulty using the MCH-FS and profiled its status and possible influencing factors among Chinese toddlers aged 1–3 years, and we found that it was significantly associated with health and development. The results showed that that feeding difficulty was prevalent in toddlers at a rate of 18%, which is close to the rates of 14–25% based on samples of general population of other countries (10, 17, 37).

As for the individual items, the highest scored item in our sample was related to bad behavior during mealtimes, such as acting up or making a big fuss (scored 3.42 ± 2.00). However, the score distribution of each of the 14 items of the MCH-FS showed variation in different countries. For example, the highest item in the normative sample in Canada was about mealtime refusal (3.14 ± 2.02) (4), but feeding distraction (3.30 ± 2.10) in Thailand (17), concern with child's eating (3.68 ± 2.05) in Brazil (18) or parent–child feeding relationship (6.54 ± 2.02) in Poland (20). In addition, the lowest average score (1.71 ± 1.21) in our sample was obtained from the item concerning the presence of gagging, spitting or vomiting, but regarding child's chewing or sucking abilities (scored around 1.33–1.49) in the other four countries mentioned above (4, 17, 18, 20). These findings demonstrated the heterogeneity of feeding problems, regardless of the good acceptability of the MCH-FS in different countries. The variance might be partly due to the existence of cultural diversity or related to some residual confounders, such as age, infant temperament, or parental cognitions.

In examining the possible influencing factors, consistent with Van Dijk's study, we found that feeding difficulty was more prominent in older children (28). A higher incidence was also reported in girls in our study, while no gender difference has been reported in previous studies (10, 17, 37). In addition, it has been suggested that a variety of prenatal and perinatal, medical, environmental, and parental factors may be related to feeding problems (38–40). However, the participants in our study were full-term healthy toddlers recruited from cities, and most of their mothers were well-educated (87.1% of them had an educational level of high school and above), with favorable levels of employment (95.9% of them were on the job) and income (54.1% of them had a monthly income higher than 5,000 RMB). In the present study, there were no differences in natal factors (delivery mode, parity, birth weight) or maternal factors (education, occupation, income) between children with and without feeding problems, however, suggesting that feeding difficulty was common even among well-developed healthy children. When it comes to early feeding behavior, a recently published systematic review showed that evidence on the association of breastfeeding duration, timing of complementary feeding introduction with parent-reported feeding difficulties were weak and inconsistent (41). Therefore, further studies should examine the variation of potential factors of feeding difficulty in broader samples.

Interestingly, we found that pre-mastication was more commonly observed among children with feeding difficulty (20.0%) than without (10.3%). Pre-mastication, as well as pre-chewing, typically refers to the behavior of caregivers who chew foods or medicines before feeding to a child (42). Despite insufficient evidence of the harms or benefits of pre-mastication on children's growth or health, it was probably used as an adjunct to ensure adequate nutrition of the infant when complementary food was introduced (43). A previous study conducted in eight cities in China by our research group indicated that caregivers using the strategy of pre-mastication might share common features of paying more attention or giving more care to their child (44). However, excess intervention by caregivers might in turn lead to the reduction in opportunities to develop children's skills in feeding themselves (45). In contrast, baby-led, rather than adult-led weaning might contribute to higher eating enjoyment, less fussy, or food picky behaviors, etc. (41, 46). Given that pre-mastication is still common in China, our result indicated that letting go, to some extent, may help to prevent feeding difficulty.

Another feeding-related factor that had a significant effect was food picky behaviors, of which the parent-reported rate in toddlers with feeding difficulty was 59.8 and 30.8% in toddlers without. Picky eating usually refers to an inadequate variety or an insufficient amount of food through rejection of food items (47). While several studies have reported the dietary intake characteristics of picky eating (47, 48), to the best of our knowledge, this is the first report regarding the role of feeding difficulty assessed by the MCH-FS on children's dietary intakes. Our study revealed that of the dimension of food groups, children with feeding difficulty had lower intakes of cereals, vegetables, and fruits. When it comes to picky eating, two recent reviews both demonstrated a common finding of reduced intake of fruits and vegetables, and fewer whole-grain products, seafood, meat, and higher savory snacks or confectionary had also been reported (47, 48). This overlap was not unexpected as feeding problems encompass a range of concerns including food selectivity (2). Of note is that although the DDS was lower for children with feeding difficulty than without, the difference was not significant. The lack of association might partly relate to the inability of one-time 24 h dietary recall to capture participants' dietary diversity adequately. Nevertheless, the daily total intakes of energy and protein were still lower in children with feeding difficulty, which suggested that the amount of food consumption might remain insufficient in children with feeding difficulty even in the presence of similar food groups. Further, it is not difficult to surmise that such avoidant or restrictive intake of specific food items would influence dietary quality, which in turn leads to poorer physical growth or health (20).

As hypothesized, we did find negative associations between high scores on the MCH-FS and a series of anthropometric Z-scores, as well as diarrhea or constipation. Consistent with our study, Rogers et al. reported that a higher MCH-FS score was negatively related to infants' weight standard deviation score (SDS) at 1 week, 1, 6, and 12 months, even though the infants who participated in their study did not have low birth weight or were not premature (10). In further analysis, the relation between feeding difficulty and HAZ was partially mediated by daily intakes of energy or protein. One possible explanation is that, children with feeding problems might show less frequency and shorter bursts of sucking as early as in the neonatal period, and continue to eat at a slower pace in toddler years (10), resulting in sustained less intake and slower height growth. Of importance is the fact that the subjects of our study were all born at term, and only around 1.5–6.5% of them had a certain kind of anthropometric Z-score under −2. This low variation might explain why the other mediation effects were not significant. Even though, our results suggested that the MCH-FS might be a useful instrument in picking up potential risk of malnutrition in a normal population. Regrettably, few studies investigated the association between feeding difficulty and health and development among normal children and there is limited ability to cross compare between countries.

In addition, we also reported for the first time that feeding difficulty was related to poorer fine motor skills and personal-social skills assessed by the ASQ-3. It is well-recognized that prolonged nutrition problems, even when not severe, may influence neurodevelopment during the critical period of infancy and toddlerhood (49). Based on a sample of 212 one-year-old normal children from Norway, Blomkvist et al. found that a higher dietary intake of fruits, vegetables or fish was positively associated with total ASQ scores (50). In addition, according to a cross-sectional survey conducted by Zhou et al. among 1,748 children aged 1–59 months in an under-developed area of China, the intake of fruits and vegetables was also related to lower risk of developmental delay (below mean minus 2 SD) in fine motor domain of ASQ (51). Therefore, we speculate that improving complementary feeding practice and dietary diversity could contribute to the neurodevelopment of infants and young children. Another point to note is that, in Zhou et al. 's study, the prevalence of developmental delay was around 21.6–31.3% (51), while in our study it was only around 1.1–3.8%, which further supports the ability of the MCH-FS to differentiate the level of development in normal samples. Conversely, worse skills on fine motor and personal-social skills might be an explanation for feeding difficulty. The fine motor development assessed by the ASQ was focused on eye–hand and hand–finger movements, coordination and pre-writing skills, and personal-social development was on self-help skills (feeding, dressing and toileting, etc.) and social interactions with others (33), which were strongly associated with the development of feeding. Thus, we suggested that additional practice of fine motor and personal-social skills could in turn help to reduce feeding difficulty.

It is important to note that feeding difficulty is common and might be transient, but it is also possible to progress to feeding disorders (39), for which a more complex and rigorous diagnostic criterion is needed. As compared to the four domains (medical, nutritional, feeding skills, and psychosocial) underlay the diagnostic criteria of PFD proposed by the ICF (1), the MCH-FS contained some overlapping items, especially in the domain of feeding skill (feeding strategies) and psychosocial (avoidance behaviors, caregiver-child relationship, etc.). In addition, the results of this study indicated that impairment in these two domains might lead to dysfunction in the nutritional domain, suggesting an interplay between domains of PFD. However, the items of the MCH-FS had limited coverage of each domain, and valid measures including but not limited to laboratory examinations are needed to improve the diagnosis of PFD.

In summary, based on multicenter data, a detailed description of feeding difficulty among Chinese toddlers aged 1–3 years was profiled and its associations with multiple health and developmental measurements were explored. Nevertheless, there are several limitations in our study. First, the cross-sectional design limited the causal inference, and some possible confounders—including infant temperamental, psychological, and environmental factors—were not included in our questionnaire. Second, multiple methods, including biological markers, which may be helpful to assess neurodevelopment were not available in the present study. Last but not least, children's feeding problems as well as their growth and development are both dynamic processes, thus further longitudinal or interventional studies are still needed.

## Conclusions

Assessed by the MCH-FS, feeding difficulty was relatively common among Chinese toddlers aged 1–3 years, and some feeding behavior such as pre-mastication might be related to its occurrence. The presence of feeding difficulty showed a negative association with normal children's health and development, which indicated the importance of early effective identification.

## Data Availability Statement

The raw data supporting the conclusions of this article will be made available by the authors, without undue reservation.

## Ethics Statement

The studies involving human participants were reviewed and approved by the Ethics Committee of Peking University. Written informed consent to participate in this study was provided by the participants' legal guardian/next of kin.

## Author Contributions

YZ, AZ, IS, and PW designed the study. ZR, AZ, CY, JZ, PL, and JL conducted the research and collected the data. AZ, IS, and HL coordinated and supervised data collection. ZR analyzed the data and drafted the initial manuscript. AZ, HL, and YZ reviewed and revised the manuscript. All authors read and approved the final manuscript.

## Funding

This research was funded by the Inner Mongolia Yili Industrial Group Co. Ltd. (No. YL20190012).

## Conflict of Interest

IS and HL are employed by the Inner Mongolia Yili Industrial Group Co. Ltd. This study received funding from the Inner Mongolia Yili Industrial Group Co. Ltd. The funder had the following involvement with the study: study design, coordination, supervision, and review and revision of the manuscript. The remaining authors declare that the research was conducted in the absence of any commercial or financial relationships that could be construed as a potential conflict of interest.

## Publisher's Note

All claims expressed in this article are solely those of the authors and do not necessarily represent those of their affiliated organizations, or those of the publisher, the editors and the reviewers. Any product that may be evaluated in this article, or claim that may be made by its manufacturer, is not guaranteed or endorsed by the publisher.
