# Supplementary material for: Feeding Difficulty Among Chinese Toddlers Aged 1–3 Years and Its Association With Health and Development
Source: Front Pediatr. 2021 Nov 23;9:758176. doi: 10.3389/fped.2021.758176 (PMC8650057; doi:10.3389/fped.2021.758176)
Supplement: Supplementary file 1 [file Data_Sheet_1.docx]

Supplementary Material

# Supplementary Tables

**Table S1.** Mean and standard deviations of the MCH-FS scores based on classification of feeding difficulty

| **Item** | **Question content** | **All subjects (n=924)** | **Feeding difficulty** | | ***P* ^a^** |
| --- | --- | --- | --- | --- | --- |
|  |  |  | **With (n=165)** | **Without (n=759)** |  |
| 1 | Difficult mealtimes | 2.76 ± 1.73 | 4.61 ± 1.49 | 2.36 ± 1.51 | ＜0.001 |
| 2 | Worries about feeding | 2.97 ± 2.01 | 4.84 ± 1.67 | 2.56 ± 1.84 | ＜0.001 |
| 3 | Poor appetite | 2.51 ± 1.53 | 4.27 ± 1.49 | 2.13 ± 1.25 | ＜0.001 |
| 4 | Start refusing food | 2.47 ± 1.53 | 3.90 ± 1.50 | 2.16 ± 1.35 | ＜0.001 |
| 5 | Long mealtimes | 2.78 ± 1.08 | 3.61 ± 1.38 | 2.60 ± 0.91 | ＜0.001 |
| 6 | Bad behavior | 3.43 ± 2.00 | 5.31 ± 1.45 | 3.03 ± 1.87 | ＜0.001 |
| 7 | Gags/spits/vomits | 1.71 ± 1.21 | 2.55 ± 1.73 | 1.53 ± 0.98 | ＜0.001 |
| 8 | Holding food in mouth | 2.74 ± 1.79 | 4.13 ± 1.80 | 2.44 ± 1.64 | ＜0.001 |
| 9 | Follow around/distract | 2.93 ± 1.94 | 4.94 ± 1.74 | 2.49 ± 1.69 | ＜0.001 |
| 10 | Force to eat | 2.35 ± 1.71 | 4.01 ± 1.81 | 1.99 ± 1.45 | ＜0.001 |
| 11 | Poor chewing | 2.22 ± 1.56 | 3.30 ± 1.82 | 1.98 ± 1.39 | ＜0.001 |
| 12 | Poor growth | 2.25 ± 1.44 | 3.52 ± 1.53 | 1.98 ± 1.26 | ＜0.001 |
| 13 | Influence parent–child relation | 1.96 ± 1.38 | 3.52 ± 1.59 | 1.62 ± 1.06 | ＜0.001 |
| 14 | Influence family relations | 2.14 ± 1.67 | 3.53 ± 1.74 | 1.83 ± 1.48 | ＜0.001 |
| Total scores | | 35.21 ± 12.90 | 56.02±6.49 | 30.69±8.88 | ＜0.001 |

^a^ *P* values were obtained from independent samples *t*-tests between children with or without feeding difficulty.

**Table S2.** Association between feeding difficulty and anthropometric Z-scores

| **variable** | **Feeding difficulty ^a^** | | **unadjusted**  ***β （95% CI）*** | **adjusted**  ***β （95% CI）*^b^** |
| --- | --- | --- | --- | --- |
|  | **with** | **without** |  |  |
| WHZ | -0.210 ± 1.013 | 0.173 ± 1.048 | **-0.382 (-0.561, -0.204)** | **-0.313 (-0.493, -0.132)** |
| HAZ | -0.122 ± 1.155 | 0.264 ± 1.086 | **-0.386 (-0.574, -0.198)** | **-0.375 (-0.565, -0.185)** |
| WAZ | -0.224 ± 1.101 | 0.284 ± 0.979 | **-0.508 (-0.677, -0.338)** | **-0.458 (-0.628, -0.288)** |
| BAZ | -0.217 ± 0.952 | 0.151 ± 1.055 | **-0.368 (-0.546, -0.190)** | **-0.313 (-0.493, -0.132)** |
| HCZ | -0.412 ± 1.228 | -0.015 ± 1.260 | **-0.397 (-0.616, -0.179)** | **-0.349 (-0.572, -0.126)** |

WHZ: weight-for-height Z-score; WAZ: weight-for-age Z-score; HAZ: height-for-age Z-score; BAZ: body mass index-for-age Z-score; HCZ: head circumference-for-age Z-score; MUACZ: mid-upper arm circumference-for-age Z-score. ^a^ Values are presented as frequency (percentage) or mean ±standard deviation. ^b^ Adjusted for age, sex, mother’s education level, household economic level, outdoor activity level, place of residence and birth weight. Bold text represents a statistically significant difference (p＜0.05).

**Table S3.** Association between feeding difficulty and illness occurrences in recent 3 months

| **variable** | **Feeding difficulty ^a^** | | **unadjusted**  ***OR （95% CI）*** | **adjusted**  ***OR （95% CI）*^b^** |
| --- | --- | --- | --- | --- |
|  | **with** | **without** |  |  |
| **Anemia** | | |  |  |
| No | 144 (92.9) | 644 (92.0) | -- | -- |
| Yes | 11 (7.1) | 56 (8.0) | 0.878 (0.427, 1.656) | 1.107 (0.508, 2.246) |
| **Respiratory diseases** | | |  |  |
| No | 73（44.8） | 357 (47.4） | -- | -- |
| Yes | 90（55.2） | 396 (52.6） | 1.111 (0.792, 1.565) | 1.078 (0.746, 1.562) |
| **Allergic diseases** | | |  |  |
| No | 133 (82.1) | 637 (86.3) | -- | -- |
| Yes | 29 (17.9) | 101 (13.7) | 1.375 (0.862, 2.140) | 1.361 (0.815, 2.217) |
| **Vomiting** |  |  |  |  |
| No | 131 (80.9) | 640 (86.6) | -- | -- |
| Yes | 31 (19.1) | 99 (13.4) | 1.530 (0.968, 2.364) | 1.359 (0.824, 2.191) |
| **Diarrhea** | |  |  |  |
| No | 114 (69.9) | 605 (81.4) | -- | -- |
| Yes | 49 (30.1) | 138 (18.6) | **1.884 (1.278, 2.751)** | **2.035 (1.323, 3.105)** |
| **Constipation** |  |  |  |  |
| No | 126 (77.8) | 633 (85.9) | -- | -- |
| Yes | 36 (22.2) | 104 (14.1) | **1.739 (1.127, 2.639)** | **2.042 (1.271, 3.241)** |

^a^ Values are presented as frequency (percentage). ^b^ Adjusted for age, sex, mother’s education level, household economic level, outdoor activity level, place of residence and birth weight. Bold text represents a statistically significant difference (p＜0.05).**Table S4.** Association between feeding difficulty and ASQ scores

| **variable** | **Feeding difficulty ^a^** | | **unadjusted**  ***β （95% CI）*** | **adjusted**  ***β （95% CI）*^b^** |
| --- | --- | --- | --- | --- |
|  | **with** | **without** |  |  |
| CM score | 50.7 ± 12.1 | 50.8 ± 11.4 | -0.065 (-2.208, 2.077) | -1.448 (-3.611, 0.715) |
| GM score | 53.3 ± 8.9 | 54.9 ± 7.8 | **-1.694 (-3.178, -0.209)** | -1.098 (-2.652, 0.457) |
| FM score | 48.1 ± 11.3 | 50.6 ± 10.1 | **-2.564 (-4.473, -0.655)** | **-2.308 (-4.326, -0.289)** |
| CG score | 49.9 ± 9.4 | 51.4 ± 9.0 | -1.495 (-3.182, 0.191) | -1.359 (-3.176, 0.458) |
| PS score | 46.0 ± 11.1 | 48.6 ± 10.0 | **-2.639 (-4.530, -0.748)** | **-2.788 (-4.807, -0.769)** |
| Total score | 247.9 ± 36.6 | 256.3 ± 30.7 | **-8.458 (-14.266, -2.549)** | **-9.000 (-15.114, -2.887)** |

CM: communication; GM: gross motor skills; FM: fine motor skills; CG: cognition; PS: personal and social skills. ^a^ Values are presented as mean ±standard deviation.  ^b^ Adjusted for age, sex, mother’s education level, household economic level, outdoor activity level, place of residence and birth weight. Bold text represents a statistically significant difference (p＜0.05).

**Table S5.** Difference of daily intakes of energy, protein and DDS with or without feeding difficulty

| **variable** | **Feeding difficulty ^a^** | | ***P* ^b^** |
| --- | --- | --- | --- |
|  | **with** | **without** |  |
| Energy (kcal) | 776.0 (611.9, 1031.6) | 888.8 (662.5, 1167.6) | **0.002** |
| Protein (g) | 27.1 (20.1, 37.6) | 31.0 (21.8, 41.6) | **0.018** |
| DDS | 5.7 ± 1.3 | 5.8 ± 1.3 | 0.229 |

DDS: dietary diversity score. ^a^ Values are presented as median (25th percentile, 50th 75th percentile) or mean ±standard deviation.  ^b^ P values were obtained from Mann-Whitney U tests or independent samples t-test. Bold text represents a statistically significant difference (p＜0.05).
